# Road mortality locations of small and medium-sized mammals along a partly-fenced highway in Quebec, Canada, 2012–2015

**DOI:** 10.1016/j.dib.2018.10.048

**Published:** 2018-10-23

**Authors:** Judith Plante, Katrina Bélanger-Smith, Ariel G. Spanowicz, Anthony P. Clevenger, Jochen A.G. Jaeger

**Affiliations:** a Concordia University Montréal, Department of Geography, Planning and Environment, 1455 de Maisonneuve Blvd. West, Suite H1255, Montréal, Québec, Canada H3G 1M8; b Concordia University Montreal, Department of Biology, 7141 Sherbrooke Street West, Montréal, Québec, Canada H4B 1R6; c Western Transportation Institute, Montana State University, Bozeman, MT, USA; d Loyola Sustainability Research Centre, Concordia University Montréal, 7141 Sherbrooke St. West, Montréal, Québec, Canada H4B 1R6

## Abstract

The data presented here consist of the locations of 839 roadkill points from four years (2012–2015) of roadkill surveys for small and medium-sized mammals (under 30 kg) from a four-lane highway in Quebec (Highway 175) during the months of May to October. Seventeen species or species groups were identified, all local to the area, and none of which were identified as species at risk, threatened, or endangered. The GPS coordinates of each roadkill event are given, along with the date, time of day (morning or evening), location (northbound or southbound lanes) and species (where possible). Within the surveyed road, 18 wildlife passages with 100 m fencing on each side of the passage entrances were built for small and medium-sized mammals. The GPS coordinates of the 18 passages and the end of each corresponding fence are also provided.

**Specifications table**

**Table:** 

Subject area	*Biology, Environmental Sciences*
More specific subject area	*Landscape Ecology, Road Ecology, Conservation Biology*
Type of data	*Excel file*
How data was acquired	*Road mortality surveys*
Data format	*Raw, cleaned*
Experimental factors	*Data were collected in the summer months only (May - October)*
Experimental features	*Road mortality surveys with cars at speed 60–70 km/h*
Data source location	*Highway 175 in Quebec, Canada*
Data accessibility	*The data are available with this article.*
Related research articles	*Plante et al.*[4]*(in press); Spanowicz et al.*[5]*(in prep.)*

**Value of the data**

• The data may be useful for studying differences in roadkill densities between fenced and unfenced areas and the fence-end effect.
• The roadkill data may be useful when studying the relationship between roadkill locations and the geometry of the road.
• It may be of interest to study the relationship between roadkill locations and landscape factors.
• The data may be useful for examining spatial patterns in road mortality (e.g., hotspots) and for comparing roadkill aggregations with analyses of landscape connectivity (such as potential movement paths predicted by circuit theory).
• Availability of several years of mortality data may be useful for a between-year comparison of the spatial patterns of road mortality.

## 1 Data

**Fig. 1:**
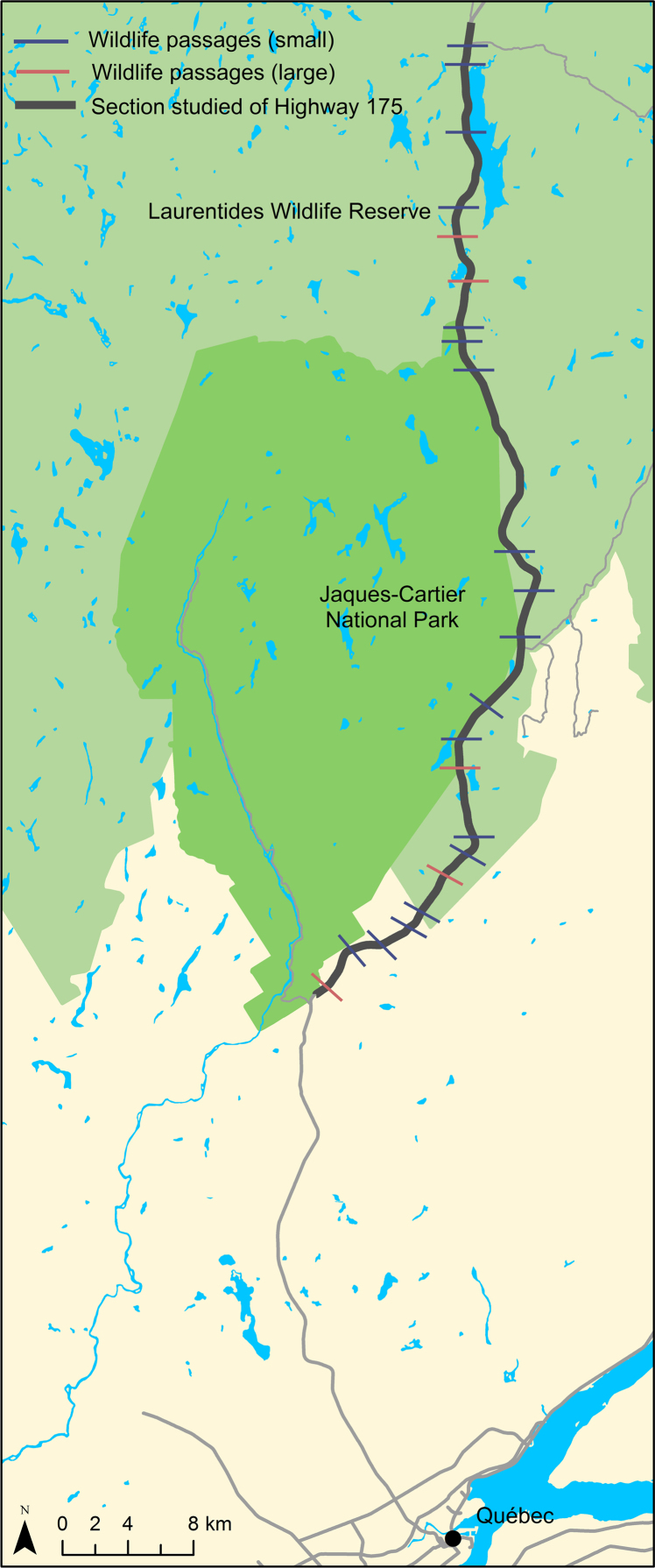
Locations of the 18 small and 5 large wildlife passages (underpasses) along the section of Highway 175 that was studied in 2012–2015 (base map: National Resources Canada). The surveyed section of the road is the section shown in dark.

The data were collected along Highway 175 (Fig. 1) between Quebec City and Saguenay as part of a project for the Ministry of Transport, Sustainable Mobility and Transportation Electrification of Quebec. The mortality data were collected by Bélanger-Smith [1] and Plante [2] and the full results are published in a report to the transport ministry in 2017 [3]. The data have also been used in Refs. [4], [5].

Highway 175 is located between Quebec City and Saguenay and borders Jacques-Cartier National Park (Parc National de la Jacques-Cartier) and the Montmorency Forest, and runs through the Laurentides Wildlife Reserve (Réserve faunique des Laurentides). The highway is located in the boreal forest biome dominated by balsam fir (*Abies balsamea*) and black spruce (*Picea mariana*). In 2014, the average annual daily traffic flow (AADT) on HW 175 was estimated 6000 vehicles (Ministère des transports du Québec 2014) [6]. Actual counts showed that the AADT was 5900 vehicles per day during the years 2011–2015. In the summer months (June-September), this average was almost 30% higher (7560 veh./day), and about 20% lower (4680 veh./day) in the winter months (December-March) (personal communication Gabriel Langevin, Ministère des transports, de la mobilité durable et de l’électrification des transports du Québec). The proportion of trucks was 15%. In the summer months, traffic volume was twice as high on Fridays and Sundays as the annual average. In 2015, the AADT was 6200 veh./day, with 7900 veh./day in the summer months and 4900 veh./day in the winter months. During the years 2005–2015, annual traffic volumes increased by 2% per year [3: App. A]. There were no projections for the future available.

**Fig. 2:**
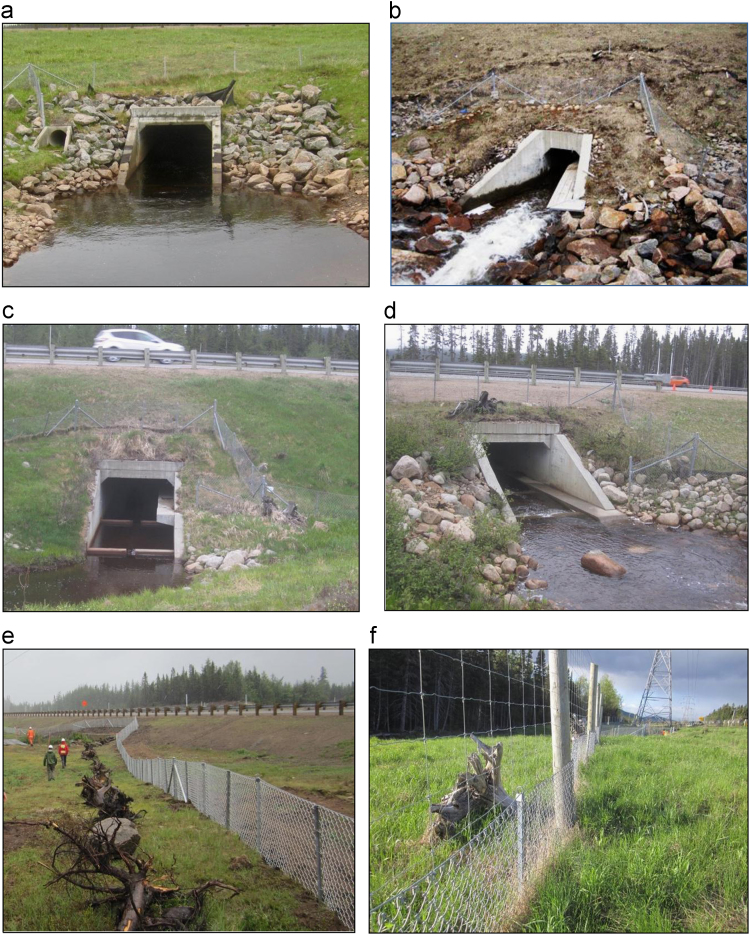
The four types of wildlife underpasses designed for small and medium-sized mammals and the two types of fences along Highway 175: (a) pipe culvert or round concrete culvert (on the left next to the water culvert; *n* = 6), (b) box culvert with a wooden ledge (*n* = 4), (c) box culvert with a concrete ledge (*n* = 7), (d) box culvert with a concrete walkway (*n* = 1), (e) fence for medium-sized mammals, and (f) combined fencing for large mammals (upper half) and medium-sized mammals (lower half) (photo credit: Concordia University).

The highway was widened from 2 to 4 lanes in 2006–2011, and during construction wildlife passages were installed with fences. Of these passages, 18 underpasses designated for small and medium-sized mammals are within the road mortality surveyed area (Fig. 1). On both sides of each entrance of these passages, exclusion fences for medium-sized species were placed. Each fence is about 100 m long on each side of the passage entrances (i.e., about 200 m in total on either side of the road) and 90 cm high with a 6 cm × 6 cm mesh size (Fig. 2).

The data provided consist of the roadkill locations from 4 summers of road mortality surveys of small and medium-sized mammals (mammals up to 30 kg) from Highway 175 in Quebec, Canada. Additionally, GPS coordinates of the locations of the 18 wildlife passages and the location of the fence ends of the 200 m fences are provided. The meaning of the column headings is as follows:

(1) The wildlife passages are named by the nearest kilometer marker (‘Passage’).
(2) The column ‘Position (km)’ provides the kilometer location along the road.
(3) Some underpasses have an open median, which is reflected in a number of 4 entrances in the column ‘Number of Entrances’ instead of 2 entrances because of the opening in the median between the northbound and southbound lanes.
(4) ‘Entrance names’ identify the entrances of the wildlife underpasses.
(5) ‘Longitude’ and ‘Latitude’ are the longitude and latitude of each entrance.
(6) ‘Entrance name (North, South, North median, South median)’ relates to the northbound and southbound lanes of Highway 175.
(7) The column ‘Large or small/med. Passage (L or S/M)’ distinguishes large (L) structures and wildlife underpasses for small and medium-sized mammals (S/M).
(8) ‘Fence names’ refer to the four sections of fence associated with each wildlife underpass.
(9) ‘Year’ refers to the year of study.
(10) ‘Session’ refers to the session of surveys (Table 3).
(11) ‘Moment of the day (Evening or morning)’ distinguishes evening surveys from morning surveys.
(12) ‘Start point (129N, 129S, 103H or 103S)’ indicates the start point of the survey along the road, which alternated among four locations.
(13) ‘Time Start’ and ‘Time End’ document the start and end times of each survey.
(14) ‘Km’ indicates a rough estimate of the location where the carcass was found based on the distance to the nearest kilometer marker along the road (as backup information in case the recorded GPS information would not make sense).
(15) ‘Longitude’ and ‘Latitude’ are the longitude and latitude of each carcass detected.
(16) ‘Species’ is the species of the carcass.
(17) ‘Direction of travel (North or South)’ distinguishes the northbound lanes from the southbound lanes (Table 1).

**Table 1:** List of species and the roadkill amounts.

**Common name**	**Scientific name**	**Number of roadkill found**
North American porcupine	*Erethizon dorsatum*	366
Red fox	*Vulpes vulpes*	47
Marmot/groundhog	*Marmota monax*	46
Mouse spp.	*Peromyscus spp.*	43
Striped skunk	*Mephitis mephitis*	42
Snowshoe hare	*Lepus americanus*	41
Shrew	*Sorex spp.*	32
Vole	*Arvicolinae spp.*	27
American red squirrel	*Tamiasciurus hudsonicus*	17
Raccoon	*Procyon lotor*	11
North American beaver	*Castor canadensis*	7
Jumping mouse	*Zapus hudsonius*	2
Canadian lynx	*Lynx canadensis*	2
Northern flying squirrel	*Glaucomys sabrinus*	2
American mink	*Neovison vison*	1
Deer mouse	*Peromyscus maniculatus*	1
American marten	*Martes americana*	1
Micromammal unknown	*Arvicolinae/Peromyscus/ Sorex spp.*	102
Mammal unknown	–	49
**Total**		839

## 2 Experimental design, materials and methods

Mortality surveys were conducted on a stretch of Highway 175 in Quebec during the summer months, May to October, for 4 years, 2012–2015. No surveys were performed during the winter months due to snowplows’ removal of roadkill.

Mortality surveys were conducted on a 136 km loop (68 km on the 2 lanes going south and 68 km on the 2 lanes going north) between km 75.5 and 143.5 of Highway 175.

The starting point of the surveys alternated between four locations to avoid potential bias that could result from using one starting point (point A =129 km N, point B = 129 km S, point C = 103km N and point D = 103km S). Surveys were conducted at an average speed of 70 km/h, with one driver and one observer, and it took approximately 3 h to complete a survey. For each carcass found the GPS coordinates, location (northbound or southbound lanes) and the species (if possible) were documented and the carcass was then removed from the road. At all times in the field, wearing a reflective/safety vest, adequate shoes and a yellow protective helmet was mandatory and while searching for roadkill or stopping to document roadkill an amber flash security light bar on the roof of the vehicle and the vehicle׳s hazard warning lights were on.

The surveys were conducted in sessions completed over 2-week intervals as shown in Table 2. The first 3 days consisted of evening surveys (Monday, Tuesday, Wednesday), then no survey on Thursday and the next 6 days consisted of morning surveys (Friday, Saturday, Sunday, Monday, Tuesday, Wednesday) and then no surveys on the last 4 days (Thursday, Friday, Saturday, Sunday). The evening surveys started three hours before sunset and the morning surveys started 30 min after sunrise.

**Table 2:** One session schedule.

	Monday	Tuesday	Wednesday	Thursday	Friday	Saturday	Sunday
Day	1	2	3	4	5	6	7
Survey time	Evening	Evening	Evening	No survey	Morning	Morning	Morning
Day	8	9	10	11	12	13	14
Survey time	Morning	Morning	Morning	No survey	No survey	No survey	No survey

Over the 4 years, a total of 34 complete sessions were performed resulting in 306 road mortality surveys (Table 3). Of these, 102 surveys were performed in the evenings, and 204 in the mornings.

**Table 3:** Number of road mortality surveys and sessions performed.

	**2012**	**2013**	**2014**	**2015**	**Total**
Number of surveys in the evening	30	27	24	21	102
Number of surveys in the morning	60	54	48	42	204
Total number of surveys	90	81	72	63	306
Number of sessions	10	9	8	7	34
Start date	June 11	June 3	May 26	June 1	–
End date	October 24	October 2	September 17	September 2	–
